# The Urinary Microbiome in Women Using Single‐Use Versus Reusable Catheters for Intermittent Catheterization: An Exploratory Substudy of the COMPaRE Trial

**DOI:** 10.1002/nau.70119

**Published:** 2025-07-24

**Authors:** Felice Emanuela Espèrance van Veen, Zhaleh Esmi Serkani, Sophie Berendsen, Robert Kraaij, Lonneke Bode, John Philip Hays, Jeroen Ronald Scheepe, Bertil Freddo Maarten Blok

**Affiliations:** ^1^ Department of Urology Erasmus University Medical Center Rotterdam the Netherlands; ^2^ Laboratory of Population Genomics, Department of Internal Medicine Erasmus University Medical Center Rotterdam the Netherlands; ^3^ Department of Medical Microbiology & Infectious Diseases Erasmus University Medical Center Rotterdam the Netherlands

**Keywords:** microbiota, sustainable development, urinary bladder, urinary catheters, urinary tract infections

## Abstract

**Aims:**

To characterize the urinary microbiome in women performing clean intermittent self‐catheterization (CISC) and explore microbial changes associated with transitioning from single‐use to reusable catheters.

**Methods:**

This microbiome study of the COMPaRE trial included female CISC patients with urinary retention randomized to either single‐use or reusable catheters. Self‐catheterized urine samples were collected at baseline and Week 6 for standard culture and 16S rRNA microbiome analysis.

**Results:**

A total of 28 patients (12 reusable, 16 single‐use) were included, with a median age of 48 years (IQR 32−60); 67.9% had neurogenic lower urinary tract dysfunction. *Escherichia‐Shigella* (36.3%) and *Lactobacillus* (22.8%) were the most prevalent genera. PERMANOVA identified significant effects of age (*p* = 0.003), menopausal status (*p* < 0.001), and catheterization cause (*p* = 0.003) on microbiome composition. Transitioning to reusable catheters was associated with significant reductions in *Escherichia‐Shigella* (*p* < 0.001) and *Aerococcus* (*p* < 0.001), and increases in *Veillonella* (*p* < 0.001) and *Finegoldia* (*p* < 0.001). No significant changes were observed in urine culture results (*p* = 0.250), alpha diversity as measured by the Shannon index (*p* = 0.862), or beta diversity as assessed by Bray‐Curtis distance (*p* = 0.096).

**Conclusions:**

*Escherichia‐Shigella* and *Lactobacillus* were the most abundant genera in the urinary microbiome of women performing CISC. Age, menopausal status, and catheterization cause significantly influenced microbiome composition. Although specific microbial shifts were observed following transition to reusable catheters, no significant changes in overall microbial diversity were detected. Larger, well‐powered studies are needed to validate these results and assess their potential clinical implications. **Trial Registration:** Clinical Trial RegistrationNL‐OMON54700/NL8296.

## 1 Introduction

Urinary retention affects millions of people worldwide and clean intermittent self‐catheterization (CISC) is the preferred treatment [1, 2]. In many high‐income countries, single‐use plastic catheters are the standard for CISC, with the average individual patient using four catheters daily resulting in approximately 1825 catheters per year. In the US, this generates around 206 million liters of waste annually—equivalent to more than 80 Olympic‐sized swimming pools [3]. With growing concerns about environmental sustainability and rising healthcare costs, reusable catheters have emerged as a potential alternative [4]. However, the safety and efficacy of reusable catheters, particularly regarding urinary tract infections (UTIs), remain uncertain due to limited quality and conflicting evidence [5]. A recent network meta‐analysis found that single‐use catheters may reduce UTI risk, but the existing studies were limited by inconsistent outcome definitions, high dropout rates, short follow‐up periods, and insufficient data on cleaning techniques and female populations [6]. To address this gap, our ongoing COMPaRE trial is assessing whether reusable catheters are as safe as single‐use catheters in terms of UTIs [7].

Recent insights into the urinary microbiome, or “urobiome,” suggest that the composition of urinary tract micro‐organisms plays a vital role in urinary health, influencing infection risk and overall bladder function [8]. This microbiome includes both commensal bacteria and potential uropathogens, creating a delicate balance that may be disrupted by procedures such as indwelling or intermittent catheterization [9, 10]. Previous studies have indicated a trend toward greater diversity in the urobiomes of patients using catheters, compared to those who void spontaneously [9, 11, 12]. In this study we aimed to characterize the urinary microbiome in women performing CISC and explore microbial differences associated with transitioning from single‐use to reusable catheters, providing foundational data that may support the clinical adoption of reusable catheters.

## 2 Materials and Methods

### 2.1 Study Design and Patient Population

This prospective microbiome study is a substudy of the COMPaRE trial, a multi‐center, randomized controlled non‐inferiority trial evaluating the safety of reusable versus single‐use catheters for CISC patients [7]. For a comprehensive description of the trial design and detailed in‐ and exclusion criteria, please refer to the published protocol [7]. In brief, CISC patients ≥ 16 years with urinary retention due to neurogenic lower urinary tract dysfunction (NLUTD) or non‐NLUTD were randomized to either the control arm (single‐use catheters) or the intervention arm (reusable catheters—Cliny or PureCath). Reusable catheters were cleaned and stored in a 1:80 dilution of 2% sodium hypochlorite and used for 2 weeks each.

A UTI was defined according to criteria of Woodford and George, based on EAU guidelines on Neurourology and the NHG guidelines for Dutch general practitioners [1, 13, 14]. A UTI requires the acute onset of specific urinary symptoms (dysuria, pain during catheterization, urinary frequency, urinary urgency, suprapubic pain, heamaturia, flank pain, fever, and delirium) combined with a positive diagnostic test (e.g., urine culture, nitrite test, dipslide, or urine sediment).

This substudy included only female patients from the Erasmus University Medical Center (Erasmus MC). In addition to the COMPaRE trial's exclusion criteria, we applied additional criteria including antibiotic treatment in the past 3 months (except for prophylaxis) and UTI symptoms at baseline. Patients provided written informed consent before sample collection. This study was approved by the Institutional Review Board of Erasmus MC (MEC‐2022‐0195).

Urine samples were collected by patients via CISC at the outpatient clinic at baseline (Week 0) and Week 6. At baseline, all patients used single‐use catheters. The single‐use group continued with single‐use catheters, while the reusable group switched to reusable catheters for the 6‐week period. During sample collection, urines were divided into two aliquots: one for standard urine culture and one for 16S rRNA sequence‐based microbiome analysis. Immediately after collection, urine samples for microbiome analysis were refrigerated at 4°C (max. 1 h) before being stored at −80°C until analysis.

### 2.2 Urine Cultures

Ten microliters of well‐mixed urine were streaked onto blood agar and MacConkey agar plates with a 10 µL inoculating loop. The plates were incubated for 2 days at 35°C in 5% CO_2_, and assessed daily for uropathogen growth. Uropathogens were identified at species level using MALDI‐ToF.

### 2.3 DNA Sequencing and Microbiome Analysis

Urine pellet samples (20–90 mL) were processed for DNA isolation using bead beating and the MagMAX Microbiome Ultra Nucleic Acid Isolation Kit on a KingFisher Flex robot. 16S rRNA amplicons targeting the V1−V3 regions were generated using a two‐step PCR method and sequenced on an Illumina NextSeq platform (2 × 300 bp). Reads were processed with FastQ and MultiQC, trimmed with TagCleaner, and analyzed using DADA2 clustering and SILVA v138.1 taxonomy. ASVs with abundance of ≥0.05% of total number of reads and present in ≥1% of samples were retained. Detailed methods are available in the Supporting Information S1: Materials.

### 2.4 Statistical Analyses

Microbial diversity and composition changes between Weeks 0 and 6 were analyzed to evaluate the impact of transitioning to reusable catheters (reusable group) versus continued single‐use catheterization (single‐use group). Alpha diversity, which indicates the richness and evenness of ASVs *within* each group, was measured using the Chao1, Shannon, and inverse Simpson indices. Changes between Weeks 0 and 6 in each study group were analyzed using the Wilcoxon signed‐rank test. Beta diversity, which compares microbial diversity *between* groups, was assessed using Bray‐Curtis dissimilarity and visualized through Principal Coordinates Analysis (PCoA). Beta diversity and differential abundance were analyzed at genus level, focusing on genera with an abundance of ≥ 0.005% of total reads and present in ≥ 10% of samples. Microbiome composition changes between Weeks 0 and 6 were evaluated using PERMANOVA (999 permutations), considering variables including: age, BMI, menopausal status, CIC frequency, and catheterization cause. Differential abundance analysis using DESeq2 was performed to identify genera and ASVs significantly associated with transitioning to reusable catheters. Patients with missing samples or those receiving antibiotics during follow‐up were excluded. Statistical significance was assessed using the Wald test, with *p* values adjusted for multiple comparisons using the Benjamini‐Hochberg correction. A corrected *p* value of < 0.01 was considered significant.

## 3 Results

In the multi‐center COMPaRE trial, 69 female patients were enrolled at the Erasmus MC, of whom 28 provided self‐catheterized urine samples for microbiome analysis (16 single‐use and 12 reusable). Patient characteristics are detailed in Table 1. The median age was 48 years (IQR 32−60), and 67.9% (19/28) had NLUTD. No significant differences in patient characteristics were found between the study groups. During the 6‐week follow‐up, one patient in the single‐use group developed a UTI at Day 25, and one patient in the reusable group at Day 21 (*p* = 0.683). Antibiotic use was similar across both groups.

**Table 1:** Patient characteristics (*n* = 28).

Characteristic	Control (*n* = 16)	Intervention (*n* = 12)	*p* value
Age, years (median [IQR])	42.0 [32.0−58.8]	52.5 [31.0−61.5]	0.371
BMI (median [IQR])	24.9 [21.5−28.9]	25.9 [21.0−28.9]	0.909
NLUTD, *n* (%)	11 (68.8)	8 (66.7)	0.612
Ethnicity, *n* (%)			
Caucasian	15 (93.8)	12 (100.0)	0.571
Latin‐American	1 (6.3)	0 (0.0)	
Hormonal status, *n* (%)			0.346
Pre‐menopausal	11 (68.8)	5 (41.7)	
Menopausal	1 (6.3)	1 (8.3)	
Post‐menopausal	4 (6.3)	6 (8.3)	
AB prophylaxis, *n* (%)	1 (6.3)	1 (8.3)	0.683
BI tap water, *n* (%)	4 (25.0)	2 (16.7)	0.479
CISC experience, years (median [IQR])	5.5 [1−15.8]	6.5 [2−11.3]	0.945
CISC frequency, *n* (%)			0.681
2−4 times/day	7 (43.8)	3 (25.0)	
5−6 times/day	5 (31.3)	4 (33.3)	
7−8 times/day	3 (18.8)	3 (25.0)	
> 8 times/day	1 (6.3)	2 (16.7)	
Spontaneous micturition, *n* (%)	9 (56.3)	6 (50.0)	0.521
UTI in 6 months before inclusion, *n* (%)	2 (12.5)	3 (25.0)	0.357
UTI during 6‐week follow‐up, *n* (%)	1 (6.3)	1 (8.3)	0.683

Abbreviations: AB = antibiotic, BI = bladder irrigation, BMI = body mass index, CISC = clean intermittent self‐catheterization, IQR = interquartile range, NLUTD = neurological lower urinary tract dysfunction, UTI = urinary tract infection.

### 3.1 Urine Cultures

A total of 87% (47 out of 54) of the urine cultures contained more than 10^2^ CFU/mL. The most common microorganisms identified were *Escherichia coli* (48%), *mixed flora* (25%) and *Klebsiella pneumoniae* (8%) (Table 2). After 6 weeks, 31% of the single‐use group and 40% of the reusable group showed changes in microorganisms. In the single‐use group, *E. coli* increased from 50% to 62.5%, while *mixed flora* decreased from 31.3% to 18.8% (*p* = 0.500). In the reusable group, *E. coli* decreased from 41.7% to 20%, and *mixed flora* increased from 16.7% to 50% (*p* = 0.250).

**Table 2:** Results of standard urine cultures at Week 0 and 6.

Patient	Control arm (*n* = 16)	
	Week 0	Week 6
1	1. *Escherichia coli* < 10^3^ CFU/mL^c^ 2. *E. coli* 10^3^−10^4^ CFU/mL^c^ 3. *Enterococcus faecalis* < 10^3^ CFU/mL	*E. coli* > 10^5^ CFU/mL
2	*E. coli* > 10^5^ CFU/mL	*E. coli* > 10^5^ CFU/mL
3	*Klebsiella pneumoniae* > 10^5^ CFU/mL	1. *K. pneumoniae* 10^4^−10^5^ CFU/mL 2. *E. coli* 10^4^−10^5^ CFU/mL
4^b^	Mixed flora 10^3^−10^4^ CFU/mL	< 10^3^ CFU/mL
5	Mixed flora 10^3^−10^4^ CFU/mL	Mixed flora 10^3^−10^4^ CFU/mL
6	*E. coli* < 10^3^ CFU/mL	*E. coli* 10^3^−10^4^ CFU/mL
7	*E. coli* > 10^5^ CFU/mL	1. *E. coli* > 10^5^ CFU/mL 2. *Proteus Mirabilis* > 10^5^ CFU/mL
8	*E. coli* 10^4^−10^5^ CFU/mL	*E. coli* 10^4^−10^5^ CFU/mL
9	*E. coli* > 10^5^ CFU/mL	*E. coli* > 10^5^ CFU/mL
10^a^	Mixed flora 10^3^−10^4^ CFU/mL	*E. coli* 10^3^−10^4^ CFU/mL
11	*E. coli* > 10^5^ CFU/mL	1. *E. coli* > 10^5^ CFU/mL *2. E. Faecalis* > 10^5^ CFU/mL
12	Mixed flora 10^3^−10^4^ CFU/mL	Mixed flora 10^3^−10^4^ CFU/mL
13	< 10^2^ CFU/mL	< 10^2^ CFU/mL
14	*E. coli* > 10^5^ CFU/mL	*E. coli* 10^4^−10^5^ CFU/mL
15	Mixed flora 10^3^−10^4^ CFU/mL	Mixed flora 10^3^−10^4^ CFU/mL
16	*Streptococcus agalactiae* 10^4^−10^5^ CFU/mL	*S. agalactiae* 10^4^−10^5^ CFU/mL

	Intervention arm (*n* = 12)	
	Week 0	Week 6
17	*E. coli* (ESBL) 10^4^−10^5^ CFU/mL	*E. coli* (ESBL) > 10^5^ CFU/mL
18	*E. coli* > 10^5^ CFU/mL	Missing urine culture
19	*E. coli* > 10^5^ CFU/mL	Missing urine culture
20	*E. coli* 10^4^−10^5^ CFU/ml	Mixed flora < 10^3^ CFU/mL
21	*E. coli* 10^4^−10^5^ CFU/mL	*E. coli* 10^4^−10^5^ CFU/mL
22^a^	*K. pneumoniae* 10^4^−10^5^ CFU/mL	Mixed flora 10^3^−10^4^ CFU/mL
23	< 10^2^ CFU/mL	< 10^2^ CFU/mL
24	< 10^2^ CFU/mL	< 10^2^ CFU/mL
25^b^	Mixed flora 10^3^−10^4^ CFU/mL	Mixed flora 10^3^−10^4^ CFU/mL
26	*K. pneumoniae* > 10^5^ CFU/mL	*S. agalactiae*/*canis* > 10^5^ CFU/mL
27	*E. faecalis* < 10^3^ CFU/mL	Mixed flora 10^3^−10^4^ CFU/mL
28	Mixed flora 10^4^−10^5^ CFU/mL	Mixed flora 10^4^−10^5^ CFU/mL

Abbreviation: CFU = colony‐forming units.

a Received antibiotics for UTI during the 6 weeks follow‐up period.

^b^ Used antibiotic prophylaxis.

^c^ Morphologically distinct microorganisms with identical name.

### 3.2 Microbiome Composition

For the microbiome analysis, one single‐use sample from Week 0 was excluded due to an outlier abundance exceeding 7 million reads, and four samples from Week 6 were missing: two patients discontinued CISC and received a suprapubic catheter or nephrostomy drains, and two samples were not collected during follow‐up. There were no significant differences in patient characteristics between those with missing samples and those with complete data.

A total of 18 618 587 quality‐filtered 16S rRNA reads were obtained from 51 samples, initially clustered into 1628 unique ASVs. After applying filters to include only ASVs with a minimum abundance of 0.005% of the total reads and presence in at least 10% of samples, 17 977 685 reads and 236 ASVs remained. In the single‐use group, the median reads per sample was 367 506 (IQR 256 978−580 806) at Week 0 and 368 924 (IQR 173 635−450 344) at Week 6. In the reusable group, the median reads per sample was 337 410 (IQR 137 117−459 561) at Week 0 and 290 043 (IQR 137 025−383 909) at Week 6.

**Figure 1:**
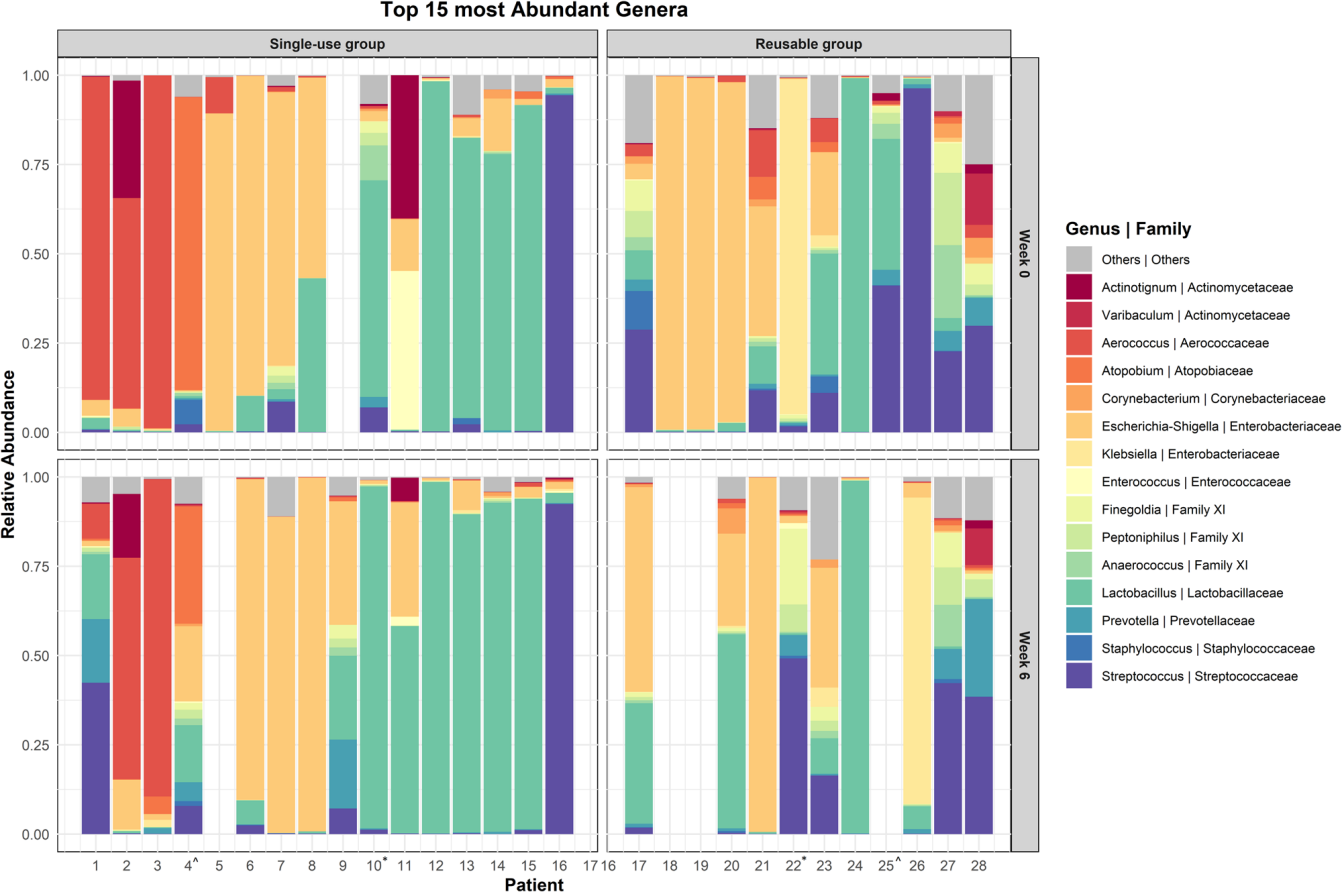
Stacked bar graph of the top 15 most abundant genera in single‐use and reusable groups at Weeks 0 and 6. Each bar represents an individual patient. *Received antibiotics for UTI during the 6 weeks follow‐up period. ^Used antibiotic prophylaxis.

The bacterial community analysis identified 18−65 genera and 56−373 ASVs per sample, with substantial variability in microbial composition among CISC patients (Figure 1). Despite this, the microbial community was dominated by a few genera, with many samples showing a single dominant genus. The most prevalent genera were *Escherichia‐Shigella* (36.3%), *Lactobacillus* (22.8%), *Aerococcus* (11.2%), *Streptococcus* (8.8%), and *Klebsiella* (7.0%). Figure 1 shows the distribution and relative abundances of the top 15 genera across individual patients, grouped by study group and time point. In the single‐use group, 66.6% of patients had a single dominant genus in both Week 0 and 6. In contrast, the reusable group showed a decrease in this proportion from 50% at Week 0 to 33.3% at Week 6 (*p* = 1.00, Fisher's exact test).

### 3.3 Alpha and Beta Diversity

**Figure 2:**
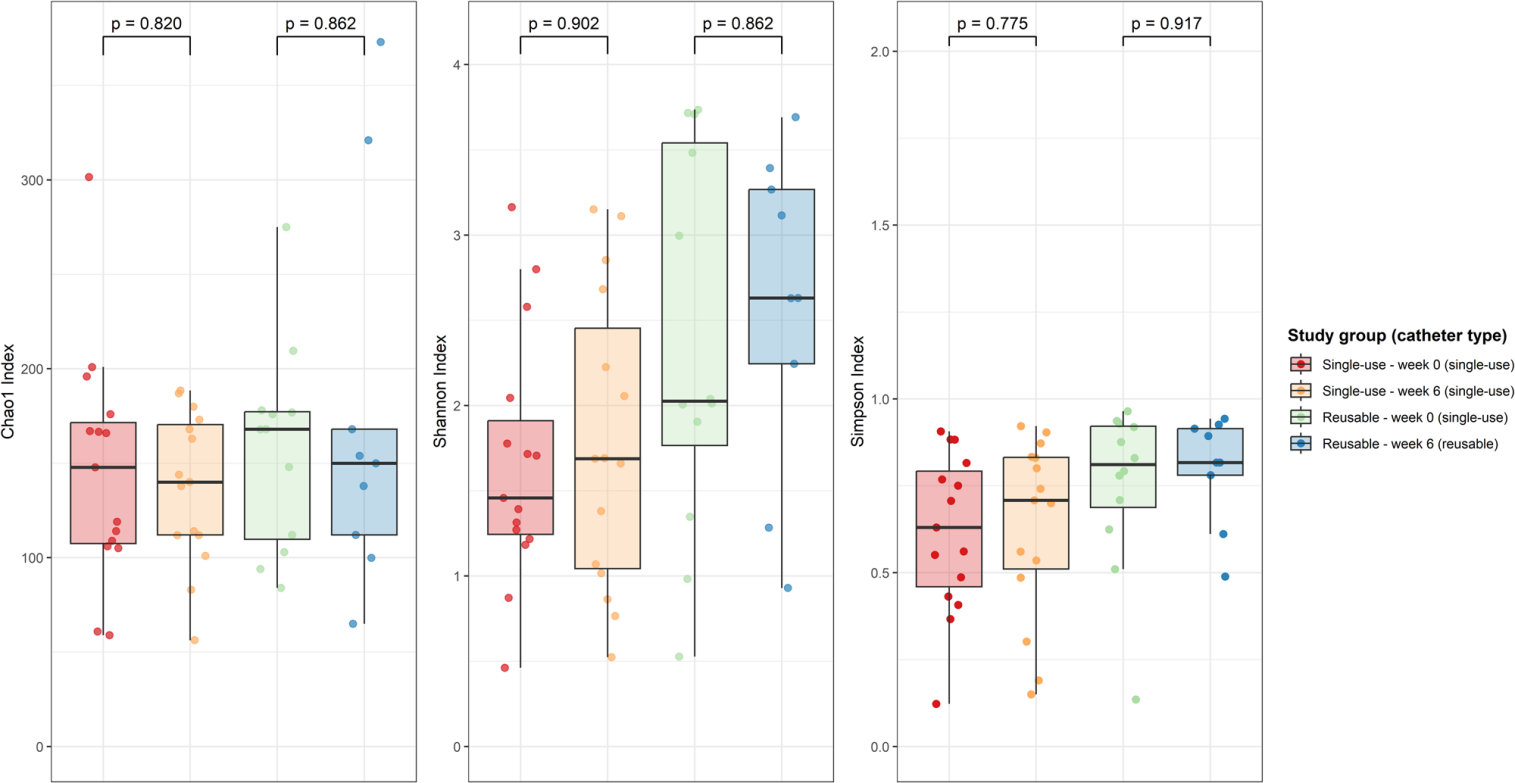
Alpha diversity box plots: Chao1 Index, Shannon Index and Inverse Simpson Index per study group (catheter type). Significance was measured using Wilcoxon signed‐rank test.

No significant changes in alpha diversity were observed over time in either study group (Figure 2). However, the Shannon index showed a non‐significant trend towards increased diversity in the reusable group, rising from 2.03 (1.77−3.54) at Week 0 to 2.63 (2.25−3.27) at Week 6, compared to a smaller increase in the single‐use group (1.46 [1.24−1.91] to 1.69 [1.04−2.45]; *p* = 0.862).

**Figure 3:**
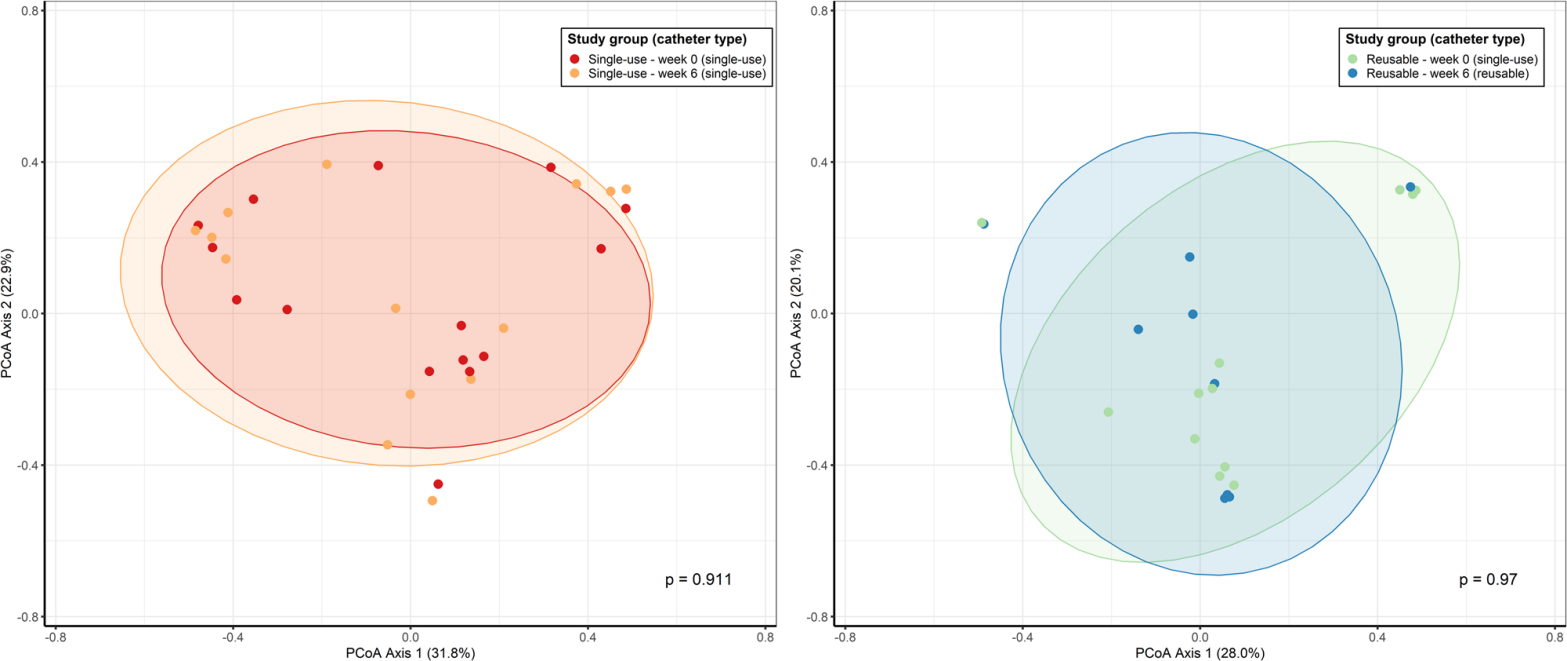
Changes in beta diversity at genus level between Weeks 0 and 6 in single‐use and reusable groups, assessed using Principal Coordinates Analysis (PCoA) of Bray‐Curtis distances. Significance was measured using PERMANOVA.

**Figure 4:**
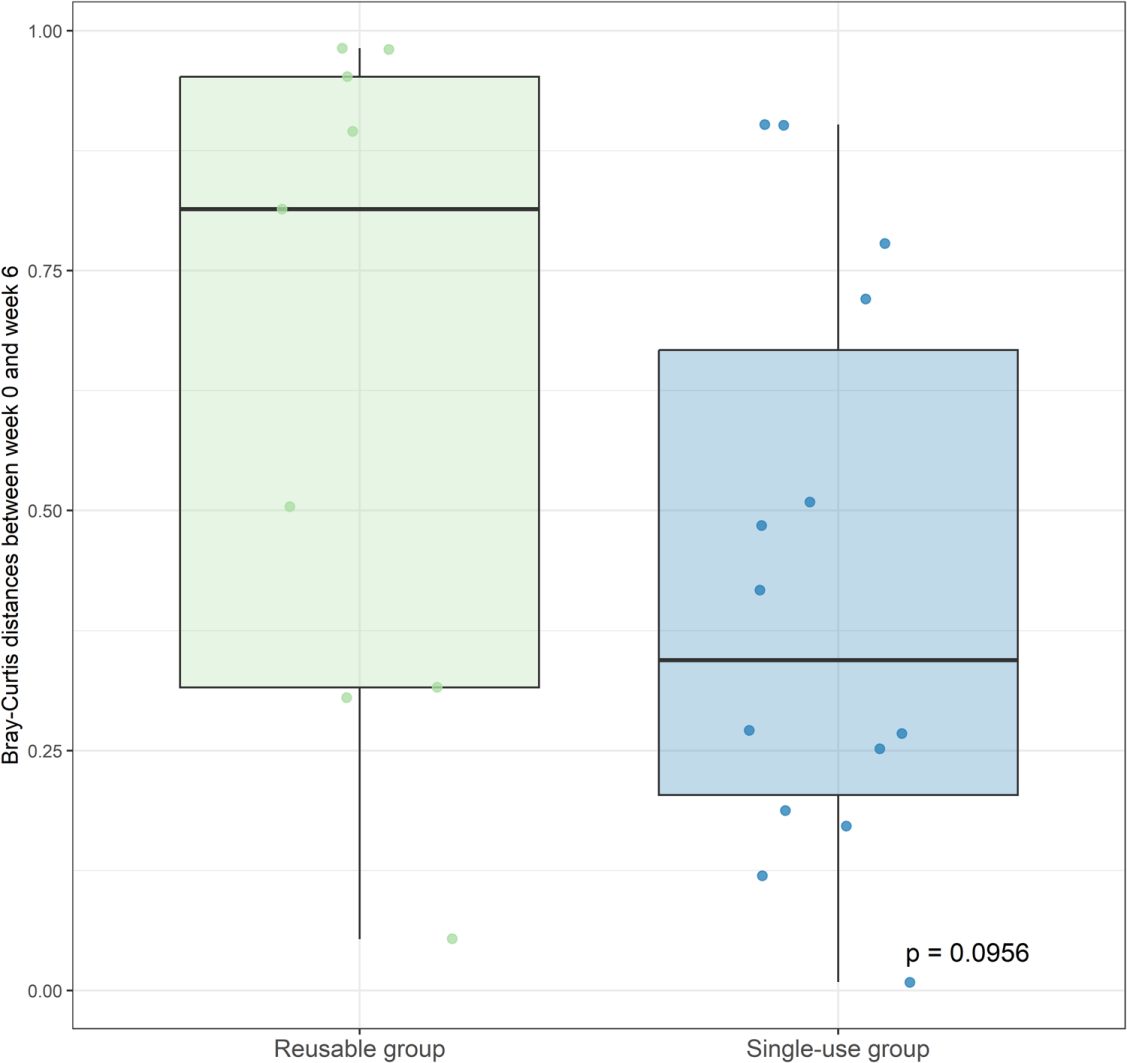
Comparison of Bray‐Curtis distances between paired samples from the same patient at Weeks 0 and 6 in the single‐use and reusable groups. The Bray‐Curtis distances were slightly larger in the reusable group indicating a greater shift in microbial composition over time. However, this difference between the two groups was not statistically significant, as measured by the Wilcoxon signed‐rank test.

Beta diversity showed no significant changes in microbiome composition in either group (single‐use: *p* = 0.907, reusable: *p* = 0.965; Figure 3). Bray‐Curtis distances between paired samples from the same patient at Weeks 0 and 6 were slightly larger in the reusable group compared to the single‐use group, though this difference was not statistically significant (*p* = 0.096, Figure 4). PERMANOVA identified significant effects of age (*p* = 0.003, *R* ^2^ = 0.056), menopausal status (*p* < 0.001, *R* ^2^ = 0.103), and catheterization cause (*p* = 0.003, *R* ^2^ = 0.052) on microbiome composition.

### 3.4 Differential Abundance Analysis

**Figure 5:**
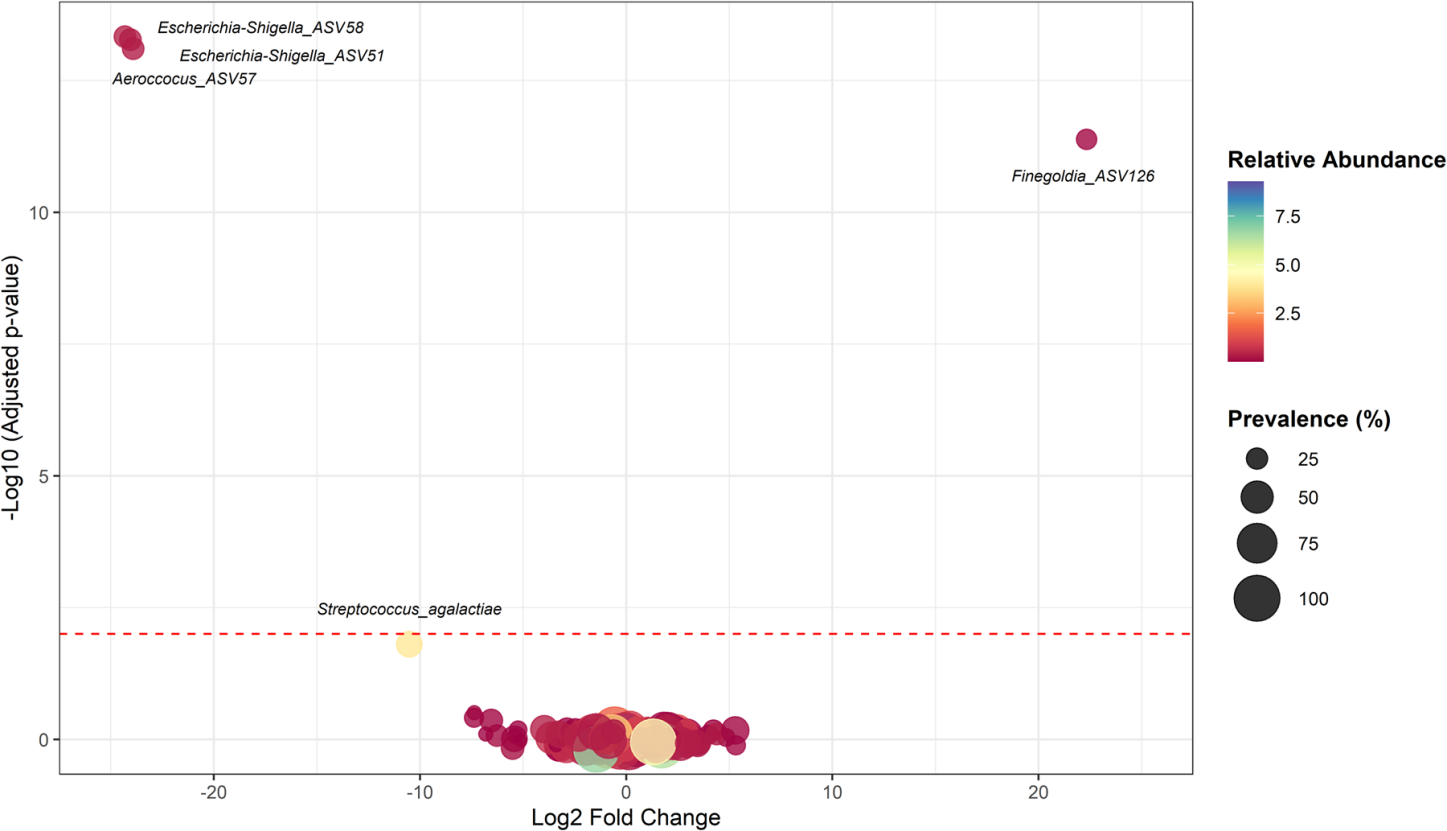
Differential abundance of ASVs. Volcano plot of estimated Log2 fold Change in ASV abundance between single‐use and reusable catheters (reusable group: Week 0 vs. 6) and corresponding Benjamini‐Hochberg corrected *p*‐values from negative binomial Wald tests as implemented in the DESeq. 2 R package. The red dashed line represents the significance threshold at −log_10_(0.01) = 2, with points above the line considered statistically significant. Four ASVs showed statistically significant differences in abundance. *Streptococcus agalactiae* (ASV17) was marginally non‐significant with a *p* value of 0.016. Prevalence indicates percentage of samples in which a given ASV is present. Relative abundance indicates mean relative abundance (%) of a given ASV.

Differential abundance analysis revealed changes over time following the transition to reusable catheters. At genus level, *Veillonella* significantly increased (*p* < 0.001). At ASV level, there was a significant decrease in abundance of unclassified *Escherichia‐Shigella* (ASV51, *p* < 0.001; ASV58, *p* < 0.001) and *Aerococcus* (ASV57, *p* < 0.001), while unclassified *Finegoldia* (ASV126) increased significantly (*p* < 0.001) (Figure 5, supporting Figure 1). A decrease in *Streptococcus agalactiae* (ASV17) approached statistical significance (*p* = 0.016). Despite these changes, the significant ASVs had overall low relative abundance (< 1.0%) and prevalence (25.5%) (Figure 5). No significant changes were observed in the single‐use group.

## 4 Discussion

In this microbiome study, we identified *Escherichia‐Shigella* and *Lactobacillus* as the most abundant urinary genera in women performing CISC. Microbiome composition was significantly influenced by age, menopausal status, and catheterization cause. Transitioning from single‐use to reusable catheters was associated with a decrease in potential uropathogens (*Escherichia‐Shigella* and *Aerococcus)* and an increase in opportunistic bacteria such as *Veillonella* and *Finegoldia*, although their overall relative abundances were low. No significant changes in overall microbiome diversity were observed, but there were patterns suggesting a shift in microbial composition, characterized by more *mixed flora*, higher alpha diversity, and slightly larger Bray‐Curtis distances in the reusable catheter group. These patterns were not statistically significant and should be interpreted with caution given the limited sample size and exploratory nature of the study.

Our findings align with previous studies, showing *Enterobacteriaceae* species as predominant in catheterizing NLUTD patients [9, 11, 12, 15]. A subset of these studies also identified *Lactobacillus* in women, with higher abundances observed in healthy controls [11, 12]. Evidence suggests that *Lactobacillus*, particularly *Lactobacillus crispartus*, plays a vital role in maintaining microbial stability and preventing uropathogen colonization [16, 17, 18]. Reduced *Lactobacillus* abundance and increased bacterial diversity are commonly associated with disease states and catheter use [11, 12, 16]. In our study, *Lactobacillus* was the second most abundant genus in women performing CISC, suggesting that CISC may help preserve *Lactobacillus* levels compared to other catheterization methods.

Reusable catheters were associated with increased abundances of *Veillonella* and *Finegoldia*. While *Veillonella* is found in healthy urinary microbiomes [8], it has also been linked to bladder cancer [19] and stress urinary incontinence [20]. Similarly, *Finegoldia magna*, frequently detected in asymptomatic women [21], has been associated with worsening urinary symptoms before pelvic surgery [22].

In addition, we observed decreased abundances of uropathogens, *Escherichia‐Shigella* and *Aerococcus*, which could indicate a reduced risk of UTIs. However, Lane et al. reported that *Enterobacteriaceae* and *Escherichia* predominate the urinary microbiome of catheterizing NLUTD patients, regardless of UTI frequency or antibiotic use [9]. Another study found that *E. coli* presence, abundance, and genomic content were not reliable UTI indicators, suggesting UTIs are more influenced by overall microbiome composition than by specific uropathogens [23].

Although there were no statistically significant changes in overall microbiome diversity, we observed patterns that may suggest subtle shifts in microbiome composition among reusable catheters users. These include a tendency toward more *mixed flora*, slightly higher alpha diversity, and increased Bray‐Curtis distances compared to single‐use catheters. Anglim et al. reported lower microbial diversity in patients with recurrent UTIs after local estrogen therapy, accompanied by fewer UTI symptoms [24]. Conversely, Wu et al. linked reduced microbial diversity to worsened lower urinary tract symptoms [25]. Taken together, these findings suggest that, although reusable catheters may alter the urinary microbiome, the clinical significance of these changes—positive or negative—requires further research.

One potential concern with reusable catheters is the increased risk of UTIs due to a higher likelihood of bacterial contamination. A study found that after an average reuse period of 21 days, 74% of catheters were contaminated with microorganisms, including 20 bacterial species. The most common were *Staphylococcus*, *Enterococcus*, and *Pseudomonas*, with biofilm formation observed in 20% [26]. Notably, most patients in this study used only soap or running water for cleaning, which likely contributed to the high contamination levels on the catheters. In contrast, Wilks et al. compared various cleaning methods for the reuse of intermittent catheters and found that the “Milton Method”, also used in our study, was particularly effective as a bactericidal agent against a wide range of uropathogens [27]. This method resulted in undetectable levels of *E. coli*, with no evidence of a viable but non‐culturable bacterial population and showed no structural damage to the uncoated PVC catheters.

In addition, it is important to note that almost all CISC patients develop bacteriuria, regardless of the technique used, as also demonstrated by our culture results. This likely results from the introduction of periurethral microorganisms into the bladder during catheterization, rather than contamination from the catheter itself [28]. Bacteriuria can induce an inflammatory response, evidenced by pyuria and elevated levels of urinary interleukins [29]. Nevertheless, more than 90% of nosocomial catheter‐associated bacteriuria cases remain asymptomatic [30], and only 20% of CISC patients with asymptomatic bacteriuria develop a UTI annually [31]. This highlights the distinction between bacteriuria and UTIs, underscoring the need for further research to clarify the clinical relevance of bacteriuria and urinary microbiome differences. While our study provides foundational insights into the urinary microbiome and its potential role in infection risk, the ongoing COMPaRE trial is expected to bridge the gap to clinical practice providing high‐level evidence on the safety of reusable catheters [7].

This study has several important limitations that should be taken into account when interpreting the findings. First, as a small exploratory substudy with only 28 participants, the limited sample size reduced the statistical power to detect significant differences between groups. No significant changes in overall microbiome diversity were observed; however, patterns suggestive of shifts in microbial composition were noted. Therefore, no definitive conclusions can be drawn from these findings. Larger, well‐powered studies are needed to validate and further explore these potential associations. Additionally, missing samples from the reusable group potentially introduced bias and reduced the robustness of our comparisons. The short follow‐up period of six weeks also restricts the ability to assess the long‐term effects of transitioning to reusable catheters on the urinary microbiome. Despite these limitations, this is the first controlled study comparing the urinary microbiome in women using single‐use versus reusable catheters for CISC, providing valuable foundational insights and underscoring the need for further research.

## 5 Conclusion

In this exploratory microbiome study, we identified *Escherichia‐Shigella* and *Lactobacillus* as the most abundant urinary genera in women performing CISC. Age, menopausal status, and catheterization cause significantly influenced microbiome composition. Transitioning from single‐use to reusable catheters was associated with a reduction in uropathogens (*Escherichia‐Shigella* and *Aerococcus)* and an increase in opportunistic bacteria (*Veillonella* and *Finegoldia)*. No significant changes in overall microbiome diversity were detected. While these specific microbial shifts may effect UTI risk, the clinical implications ‐ positive or negative ‐ remain unclear. Future research should validate these findings in larger cohorts and evaluate the clinical impact of microbiome changes on UTI risk.

## Ethics Statement

This study was approved by the Institutional Review Board of the Erasmus MC, Rotterdam, the Netherlands (MEC‐2022‐0195).

## Consent

Patients provided written informed consent before the study.

## Conflicts of Interest

The authors declare no conflicts of interest.

## Supporting information


**Supplementary Figure 1:** Log2‐transformed relative abundance of bacterial ASV's between week 0 and week 6 of the reusable group (single‐use vs. reusable catheter).

**Supplementary Methods**: DNA Sequencing and Microbiome Analysis.

**supmat**.

## Data Availability

The data that support the findings of this study are available from the corresponding author upon reasonable request.
