# Supplementary figures and images for: The Urinary Microbiome in Women Using Single‐Use Versus Reusable Catheters for Intermittent Catheterization: An Exploratory Substudy of the COMPaRE Trial
Source: Neurourol Urodyn. 2025 Jul 24;44(7):1474–83. doi: 10.1002/nau.70119 (PMC12319515; doi:10.1002/nau.70119)

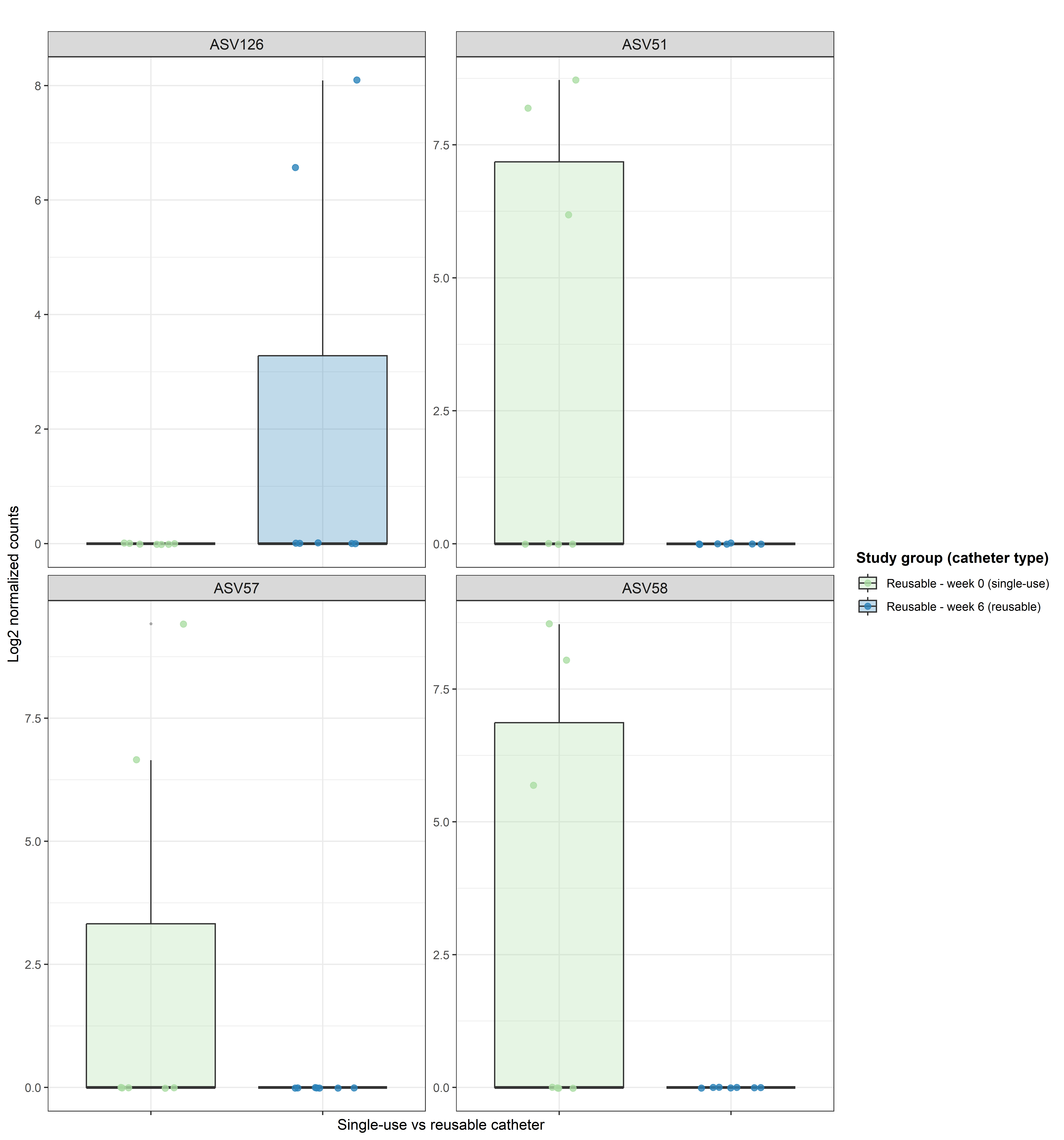

Supplement: Supplementary file 1 — Supplementary Figure 1: Log2‐transformed relative abundance of bacterial ASV's between week 0 and week 6 of the reusable group (single‐use vs. reusable catheter). [file NAU-44-1474-s002.tiff]
