# Supplementary material for: The Urinary Microbiome in Women Using Single‐Use Versus Reusable Catheters for Intermittent Catheterization: An Exploratory Substudy of the COMPaRE Trial
Source: Neurourol Urodyn. 2025 Jul 24;44(7):1474–83. doi: 10.1002/nau.70119 (PMC12319515; doi:10.1002/nau.70119)
Supplement: Supplementary file 2 — Supplementary Methods: DNA Sequencing and Microbiome Analysis. [file NAU-44-1474-s003.docx]

**Supplementary methods**

DNA Sequencing and Microbiome Analysis

DNA isolation, amplicon generation and sequencing were performed by the Genomics Core Facility of the Erasmus MC. Urine pellet samples (20-90 mL of urine, centrifuged at 4165 rcf for 10 minutes and resuspended in the remaining 1ml urine) were kept at -80°C. 300ul of urine pellet was bead beated (MagMAX™ Microbiome Bead Tubes; Thermo Fisher scientific, Waltham, MA, USA) using a MagNA Lyser instrument (Roche Diagnostics, Almere, The Netherlands) at 7,000 rpm for 45 seconds. Samples were centrifuged at 6,000xg for 5 min and 400ul of supernatant was subjected to automated DNA isolation using the MagMAX™ Microbiome Ultra Nucleic Acid Isolation Kit (Thermo Fisher scientific, Cat. No.: A42358) according to the manufacturer's protocol setting ’High throughput isolation of Nucleic Acid (RNA and DNA) from soil, biofluids, and other samples’. The isolation was performed on a KingFisher Flex robot (Thermo Fisher scientific). 16S rRNA amplicon generation was performed using an Illumina Nextera XT based 2-steps PCR method using the Roche KAPA HiFi HotStart ReadyMix (2X; Roche Life Sciences, Penzberg, Germany, Cat. No.: 09420711001) and appropriate cross contamination (DNA-free water), positive (Zymo biomics; Zymo Community Mock (art. No. D6306) & Zymo Gut Microbiome Standard (art. No. D6331)) and negative controls (DNA-free water). The V1-V3 variable regions of the bacterial 16S rRNA gene were amplified using primers 8F 5’- AGAGTTTGATCCTGGCTCAG-3’ and 518R 3’- CGTATTACCGCGGCTGCTG-5’. PCR products were cleaned using the Ampure XP beads protocol (Beckman Coulter, Brea, CA, USA). DNA concentrations were measured at various points during wet-lab processing using the QuantiFluor dsDNA kit (Promega, Madison, WI, USA) on a VarioSkan LUX multiplate reader (Thermo Fisher scientific, Waltham, MA, USA). Finally, amplicon sequencing was performed on an Illumina NextSeq™1000/2000 P1 (600 cycles) PE300 Flowcell (San Diego, CA, USA) platform at 2 x 300bp read length using ExAmp chemistry.

After sequencing, read quality was assessed using FastQC and MultiQC.^1, 2^ Primer sequences were trimmed using TagCleaner (version 0.16).^3^ Cleaned reads were entered into DADA2 (version 1.32.0) analysis using filter settings: truncQ=2, maxEE_F=2, maxEE_R=2, maxN=0 and trimRight=0.^4^ Amplicon Sequence Variants (ASVs) were assigned a taxonomy using the Ribosomal Database Project naïve Bayesian classifier with SILVA v138.1 ribosomal database.^5^ The resultant ASV taxonomy table was imported into phyloseq (1.48.0) to create a phyloseq object.^6^ Taxa were filtered based on a minimum abundance of at least 0.05% of total number of reads and presence in at least 1% of samples. A phylogenetic tree was generated within phyloseq object using Phangorn (version 2.11.1).^7^

**Method references**

1. Andrews S. FastQC: A Quality Control Tool for High Throughput Sequence Data. Available from: <http://www.bioinformatics.babraham.ac.uk/projects/fastqc/>. Accessed [3 June 2024].

2. Ewels, P., Magnusson, M., Lundin, S., Kaller, M.: MultiQC: summarize analysis results for multiple tools and samples in a single report. Bioinformatics, **32:** 3047, 2016

3. Schmieder, R., Lim, Y. W., Edwards, R.: Identification and removal of ribosomal RNA sequences from metatranscriptomes. Bioinformatics, **28:** 433, 2012

4. Callahan, B. J., McMurdie, P. J., Rosen, M. J. et al.: DADA2: High-resolution sample inference from Illumina amplicon data. Nat Methods, **13:** 581, 2016

5. Quast, C., Pruesse, E., Yilmaz, P. et al.: The SILVA ribosomal RNA gene database project: improved data processing and web-based tools. Nucleic Acids Res, **41:** D590, 2013

6. McMurdie, P. J., Holmes, S.: phyloseq: an R package for reproducible interactive analysis and graphics of microbiome census data. PLoS One, **8:** e61217, 2013

7. Schliep, K. P.: phangorn: phylogenetic analysis in R. Bioinformatics, **27:** 592, 2011
