# Supplementary material for: The Urinary Microbiome in Women Using Single‐Use Versus Reusable Catheters for Intermittent Catheterization: An Exploratory Substudy of the COMPaRE Trial
Source: Neurourol Urodyn. 2025 Jul 24;44(7):1474–83. doi: 10.1002/nau.70119 (PMC12319515; doi:10.1002/nau.70119)
Supplement: Supplementary file 3 — supmat. [file NAU-44-1474-s001.docx]

**Supplementary**

**Figure 1.** Log2-transformed relative abundance of bacterial ASV’s between week 0 and week 6 of the reusable group (single-use vs. reusable catheter). Each dot represents an individual sample. *Finegoldia_ASV126* showed a significant increase in abundance (p < 0.001), while *Escherichia-Shigella_ASV51, Aerococcus_ASV57* (p < 0.001), and *Escherichia-Shigella_ASV58* (p < 0.001) showed significant decreases. Significance was measured using the Wald test.
